# Calcitriol Inhibits Hedgehog Signaling and Induces Vitamin D Receptor Signaling and Differentiation in the *Patched* Mouse Model of Embryonal Rhabdomyosarcoma

**DOI:** 10.1155/2012/357040

**Published:** 2012-02-21

**Authors:** Anja Uhmann, Hannah Niemann, Bérénice Lammering, Cornelia Henkel, Ina Heß, Albert Rosenberger, Christian Dullin, Anke Schraepler, Walter Schulz-Schaeffer, Heidi Hahn

**Affiliations:** ^1^Institute of Human Genetics, University of Goettingen, 37073 Göttingen, Germany; ^2^Department of Genetic Epidemiology, University of Goettingen, 37073 Göttingen, Germany; ^3^Department of Diagnostic Radiology, University of Goettingen, 37073 Göttingen, Germany; ^4^Central Animal Facility, University of Goettingen, 37073 Göttingen, Germany; ^5^Department of Neuropathology, University of Goettingen, 37073 Göttingen, Germany

## Abstract

Rhabdomyosarcoma (RMS) is the most common soft tissue sarcoma in children. Aberrant Hedgehog (Hh) signaling is characteristic of the embryonal subtype (ERMS) and of fusion-negative alveolar RMS. In the mouse, ERMS-like tumors can be induced by mutations in the Hh receptor Patched1 (Ptch). As in humans these tumors show increased Hh pathway activity. Here we demonstrate that the treatment with the active form of vitamin D_3_, calcitriol, inhibits Hh signaling and proliferation of murine ERMS *in vivo* and *in vitro*. Concomitantly, calcitriol activates vitamin D receptor (Vdr) signaling and induces tumor differentiation. In addition, calcitriol inhibits ERMS growth in *Ptch*-mutant mice, which is, however, a rather late response. Taken together, our results suggest that exogenous supply of calcitriol could be beneficial in the treatment of RMS, especially in those which are associated with aberrant Hh signaling activity.

## 1. Introduction

The Hh signaling pathway is involved in the regulation of proliferation and differentiation of different cell types, tissues, and organs [1]. In the absence of Hh, Ptch suppresses the activity of its signaling partner Smoothened (Smo). Hh pathway activation is initiated when Hh ligands bind to Ptch, thereby relieving its inhibition on Smo. This results in the transcription of Hh target genes including *Gli1* [1]. Aberrant Hh signaling caused by inactivating *Ptch *mutations, activating *Smo* mutations, or Hh overexpression leads to tumor formation [2].

RMS is a myogenic tumor and the most common soft tissue sarcoma in children. RMS occurs as 2 major subtypes, which are ERMS and alveolar RMS (ARMS). ARMS occurs in adolescents and young adults. Seventy-five percent of ARMS are characterized by chromosomal translocations resulting in fusion genes consisting of the DNA-binding domain of the PAX7 or PAX3 and the transactivation domain of the forkhead transcription factor FOXO1 [3]. Approximately 25% of ARMS lack this translocation and are thus fusion negative, despite classical alveolar histology [4, 5]. ERMS is the more frequent RMS subtype and accounts for two-thirds of all RMS. It predominantly occurs in infants and young children [3, 6].

Therapies of RMS are typically multidisciplinary and combine complete surgical excision or local irradiation with chemotherapy. The 5-year overall survival for RMS is approximately 73% for ERMS and 48% for ARMS [7]. However, the survival rate for metastatic disease is only 10–30% for ARMS [8] and approximately 40% for ERMS [9]. This shows the need for new treatment options especially for patients with recurrent or metastatic RMS.

We and others recently showed that sporadic ERMS and fusion-negative ARMS overexpress Hh target genes [10, 11]. This finding has important implications for molecular targeted therapies in these subtypes, because they may be sensitive to a treatment with Hh pathway inhibitors.

Mice heterozygous for the Hh receptor Ptch develop embryonal subtype-like RMS [12–14]. Therefore, these mice present a suitable model for the preclinical evaluation of Hh pathway antagonists in the treatment of ERMS, in which Hh signaling is active. We recently showed that the specific Smo-inhibitor cyclopamine does not exert any antitumor effect in this mouse model, although it partially suppressed Hh pathway activity [15]. This implies that Smo-independent events may contribute to progression of Hh-associated RMS. Thus, therapy of Hh-associated RMS may require targeting of additional signaling pathways.

Vitamin D_3_ and its derivatives are known to have antiproliferative effects on different cancers and cancer cell lines including the RMS cell line HS729 [16, 17]. Until recently, the antitumoral effects of vitamin D_3_ were solely explained by binding of the biologically active form of vitamin D_3_, calcitriol (1*α*,25(OH)_2_D_3_), to the Vdr and the subsequent regulation of Vdr-bound genes. This so-called “genomic” calcitriol/Vdr-signaling can be monitored by the transcription of *Cyp24a1*. In addition, calcitriol elicits rapid, so-called “nongenomic” (i.e., transcription-independent) effects such as calcium influx.

Importantly, we recently demonstrated that calcitriol inhibits growth of basal cell carcinoma (BCC), which is an Hh/Ptch-driven tumor [18]. In BCC, the antitumoral property of calcitriol is accompanied by activation of the Vdr signaling pathway, by induction of differentiation and by simultaneous inhibition of Hh signaling activity [18]. The latter effect is in line with data published by Bijlsma et al. who provided first evidence that vitamin D_3_ inhibits the Hh pathway at the level of Smo [19].

The crosstalk between calcitriol/Vdr and Hh pathways prompted us to investigate the effect of calcitriol on Vdr and Hh signaling, growth, apoptosis, and differentiation of Hh-associated RMS* in vitro* and *in vivo* using the *Ptch* ^neo67/+^ mouse model for ERMS [12].

## 2. Material and Methods

### 2.1. Compounds

Calcitriol (Sigma-Aldrich, Germany) and cyclopamine (Toronto Research Chemicals Inc., Canada) were dissolved in ethanol (EtOH). Final concentrations for *in vitro* experiments are indicated in the respective experiments and correspond to those normally used in culture [16, 20, 21]. For *in vivo* use, calcitriol was diluted individually for each animal in 20 *μ*L EtOH/1200 *μ*L sterile sunflower oil (Sigma-Aldrich) to obtain a final concentration of 50 ng/kg in 50 *μ*L.

### 2.2. Animals and Treatment of Tumor-Bearing Ptch^neo67/+^ Mice with Calcitriol

Heterozygous *Ptch* ^neo67/+^ mice were maintained on a mixed C57BL/6 × Balb/c background, which renders them susceptible for ERMS-like tumors [12, 22]. The tumors are deficient in *Ptch* and show a strong Hh signaling activity [23, 24].

ERMS-bearing *Ptch* ^neo67/+^ mice were subjected to VCT (volumen computer tomography) analysis (see below). Animals with similar tumor volumes were selected and randomized into two groups. After randomization, mice were injected daily i.p. with either 50 ng/kg/d calcitriol or vehicle for 8 weeks. 50% of the animals of each group were randomly euthanized directly after treatment. The remaining mice were observed for additional 4 weeks. Tumors were dissected 24 h after the last calcitriol injection or after the medication-free period and were used for molecular analyses.

Mice were fed with calcium- and phosphate-reduced and vitamin D_3_-free food (ssniff Spezialdiaeten, Soest, Germany; E15312-14) [25] one week before and during the injection period and for one week thereafter.

All animals were treated and housed in accordance with the German animal protection law.

### 2.3. Measurement of Tumor Size

Imaging of tumors was performed using a laboratory animal flat panel volume computer tomography (GE Global Research; USA) as described [15, 26].

### 2.4. Analysis of Calcium Blood Serum Values

100 *μ*L of blood were collected from the retroorbital plexus. Serum calcium concentrations were measured using an O-cresolphthalein-based assay (cobas, Roche Diagnostics GmbH, Germany).

### 2.5. Histopathology and Immunohistochemistry

ERMS and normal skeletal muscle (SM) of *Ptch* ^neo67/+^ mice were embedded in paraffin for histological analyses or were used for isolation of total RNA (see below). The identity of ERMS was confirmed by examination of H- & E-stained sections. Paraffin sections were stained using an anti-Ki67 and antiactive caspase 3 antibody as described [15].

### 2.6. Cell Lines and Primary Cell Culture of RMS

Primary cultures derived from ERMS of *Ptch* ^neo67/+^ mice were established as published previously [15].

### 2.7. Cell Culture Experiments

For gene expression analysis and BrdU incorporation or Caspase 3 and 7 (effector caspases involved in apoptosis) assays, 100,000 and 4,000 cells/well were seeded in 6-well and 96-well plates, respectively. After 24 h, the cells were washed and incubated for additional 48 h with medium supplemented with 10 nM calcitriol, 5 *μ*M cyclopamine, or ethanol.

Cell proliferation was measured after BrdU-pulsing for the last 22 h using a cell proliferation BrdU ELISA (Roche Diagnostics GmbH).

Activity of Caspase 3 and 7 was measured using the Caspase-Glo 3/7 Assay (Promega) according the manufacturer's instructions.

Data shown are representative for at least three independent experiments each performed in triplicate.

### 2.8. Reverse Transcription and Quantitative RT-PCR Analyses

Total RNA was extracted using TriReagent (Sigma-Aldrich). Synthesis of cDNA and primer combinations for amplification of *18S r*RNA, *Cyp24a1, Vdr, Gli1, MyoD1, p27*, and* MRF4* transcripts used for quantitative real-time PCR (qRT-PCR) were described previously [15, 18, 27]. Amplification of *18S r*RNA was performed as an endogenous control for the normalization of target gene expression. The amount of target and endogenous reference was determined using the relative standard curve method. Each sample was measured in triplicates.

### 2.9. Statistics

Mann-Whitney-U testing was performed to determine the significance of the results from qRT-PCR-analyses, BrdU incorporation assays, caspase 3/7 activity assays, Ki67 counts, and calcium serum concentrations.

ANOVA models were fitted to test for differences in the tumor volumes (logarithmic transformed) between treatment at baseline and during follow-up groups. Tukey's method was used to adjust for multiple testing. The global level of significance was set to 5.

## 3. Results and Discussion

**Figure 1:**
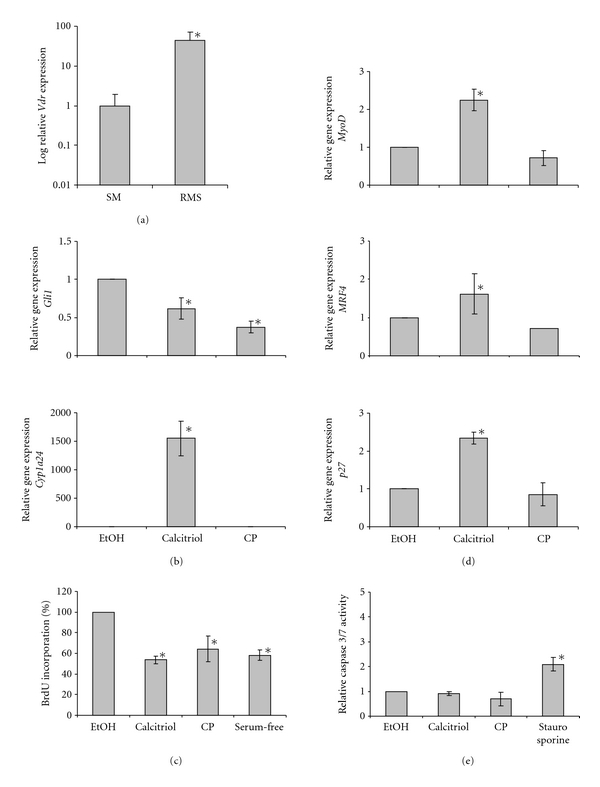
Calcitriol inhibits Hh signaling and proliferation and induces Vdr signaling and differentiation of primary ERMS cultures of *Ptch* ^neo67/+^ mice. (a) *Vdr* expression levels of ERMS (*n* = 8) compared to normal skeletal muscle (SM; *n* = 8). (b) *Gli1* and *Cyp24a1* expression levels and (c) BrdU incorporation of primary ERMS cultures after treatment with vehicle (EtOH), calcitriol, or cyclopamine (CP). (d) *MyoD1*, *MRF4*, and *p27* expression levels and (e) activity of caspase 3 and 7 of primary ERMS cultures after treatment with EtOH, calcitriol or CP. Values of vehicle-treated controls for *Gli1, MyoD1*, *MRF4*, and *p27* expression were set to 1. Expression levels were normalized to the expression of *18S r*RNA gene. For BrdU incorporation or caspase 3/7 assays, cells treated with serum-free medium or 500 nM staurosporine (Sigma-Aldrich) served as positive controls, respectively. The values of BrdU incorporation and caspase 3/7 activity represent the percentage of respective vehicle-treated control, which was set to 1. Asterisks: *P* < 0.05; error bars: mean ± SD.

ERMS of *Ptch*-mutant mice showed high levels of *Vdr* transcription (Figure 1(a)). This is similar to human RMS cell lines, which highly express *VDR* [17]. Short-term cultures of murine ERMS revealed that treatment with 10 nM calcitriol increased the expression of the Vdr target gene *Cyp24a1*, whereas it downregulated the expression of the Hh target gene *Gli1* (Figure 1(b)). As expected, treatment of the cells with 5 *μ*M of the specific Hh-antagonist and Smo-inhibitor cyclopamine did not activate Vdr signaling (Figure 1(b)), but strongly inhibited Hh signaling activity (Figure 1(b)).

To assess the antiproliferative effect of calcitriol, BrdU-incorporation assays were conducted. Calcitriol efficiently inhibited the proliferation of ERMS primary cultures, and its anti-proliferative effect was comparable to that of cyclopamine (Figure 1(c)).

In addition, calcitriol induced a significant increase in the expression of the muscle differentiation markers *MyoD*,* MRF4*, and *p27* (Figure 1(d); [28, 29]). The induction of muscle differentiation was specific for calcitriol and was not seen with cyclopamine.

The before-mentioned effects were not accompanied by activation of caspase 3/7 activity (Figure 1(e)).

These data show that calcitriol inhibits proliferation of ERMS cells* in vitro*. This is similar to cyclopamine, which likewise blocks proliferation of murine (see Figure 1(c)) and human ERMS cell lines [30]. In addition, calcitriol induces* MyoD* expression. This is specific for calcitriol, because cyclopamine did not change *MyoD *levels, although Hh signaling has been reported to regulate the expression of this gene [31]. Similarly calcitriol, but not cyclopamine, induces expression of *MRF4* and *p27*, both of which drive cell cycle exit and differentiation of myoblasts [32, 33]. Since calcitriol-mediated regulation of muscle growth and differentiation is either calcium or Vdr related [34–36], the calcitriol-induced expression of muscle differentiation markers in ERMS is likely due to one of these mechanisms.

Together, our *in vitro* analyses demonstrate that calcitriol inhibits Hh signaling activity and cell proliferation of Hh-associated ERMS in a similar manner when compared to cyclopamine. However, in contrast to cyclopamine, calcitriol additionally induces Vdr signaling and differentiation of the tumors.

**Figure 2:**
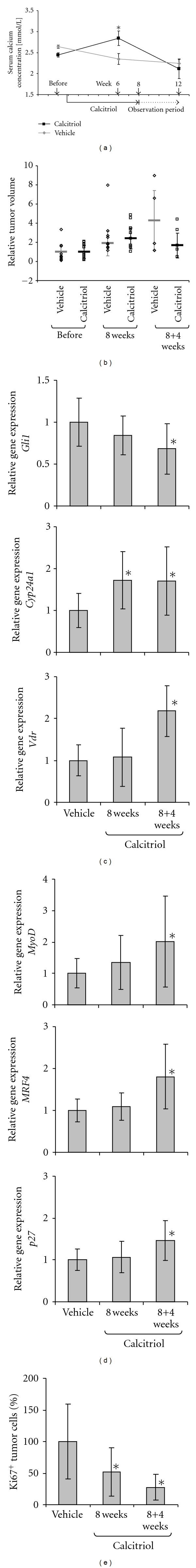
Calcitriol inhibits Hh signaling and proliferation and induces Vdr signaling and differentiation of ERMS in *Ptch* ^neo67/+^ mice *in vivo*. (a) Calcitriol treatment scheme and calcium serum concentrations of ERMS-bearing *Ptch* ^neo67/+^ mice. Animals were either sacrificed directly at therapy end (8 weeks of treatment) or after an additional 4-week observation period (dashed arrow). (b) Tumor volume was determined by VCT analysis before therapy (before), at the end of therapy (8 weeks), and 4 weeks thereafter (8 + 4 weeks). Given is the relative median tumor size (horizontal bars) for each time point, the individual tumor volumes (dots), and the standard deviation of tumor volumina. ERMS treated with calcitriol (black) or vehicle (grey). Median tumor volume before onset of treatment was set as 1. Median of the absolute tumor volumes at time point of randomization was 0.011 cm^3^ ±SEM 0.0033. (c) *Gli1*, *Cyp24a1*, and *Vdr*, (d) *MyoD1*, *MRF4*, and *p27* expression levels and (e) percentages of Ki67^+^ tumor cells/all tumor cells of ERMS after calcitriol-treatment for 8 weeks (8 weeks) or after the additional 4-week observation period (8 + 4 weeks) compared to vehicle-treated tumors (vehicle). Expression levels were normalized to the expression of *18S r*RNA gene. Values of vehicle-treated controls for *Gli1*, *Vdr, MyoD1, MRF4*, and *p27* expression were set to 1. Values of vehicle-treated controls for Ki67^+^ tumor cells were set to 100%. Asterisks: *P* < 0.05; error bars: mean ± SD.

Next, we assessed the *in vivo* effects of calcitriol in the *Ptch* ^neo67/+^ mouse model for ERMS. ERMS-bearing mice were randomly divided into two groups. Tumor volumes of these animals at the beginning of the study did not significantly differ within each group and among groups (tested by ANOVA). Mice in the treatment group (*n* = 14) obtained a daily dose of 50 ng/kg calcitriol over a period of 8 weeks, whereas animals in the control group were treated with vehicle (*n* = 10). At the end of the therapy, tumor volume of all animals was measured by VCT analysis, and 7 calcitriol- and 4 vehicle-treated mice were sacrificed for molecular tumor analysis. To detect delayed effects of the treatment, the remaining 7 and 6 animals, respectively, were observed for 4 additional weeks (observation period in Figure 2(a)), subjected to VCT and euthanized thereafter.

Calcitriol therapy led to a significant and reversible increase in serum calcium concentrations (Figure 2(a)), without causing weight loss, hypercalcemia-driven kidney damage, or signs of nephrocalcinosis (data not shown). 

In Figure 2(b) fold increase of tumor volumes of the calcitriol- or vehicle-treated mice is shown. The volumes of the majority of RMS increased irrespective of treatment, and at the end of the 8-week treatment period the average overall tumor growth rate was not different between the cohorts (Figure 2(b); 8 weeks). In the medication-free observation time, the tumors of the control group grew steadily and on average enlarged more than 4-fold over their baseline size (Figure 2(b); 12 weeks). In contrast, tumors treated with calcitriol remained stable. The difference between the cohorts was however not significant.

As already observed in cell culture, the *in vivo* calcitriol treatment resulted in an activation of Vdr signaling. This was revealed by a significant induction of *Cyp24a1* transcription in the tumors (Figure 2(c)). *Cyp24a1* induction was seen directly after the 8-week treatment period and was sustained in the 4-week medication-free period. The induction of *Cyp24a1* is consistent with the preexisting expression of the calcitriol receptor Vdr in ERMS (see Figure 1(a)). We also observed an increase in *Vdr* expression, which was becoming significant after the medication-free period (Figure 2(c)). Thus, *Vdr* induction seems to be a late effect of the calcitriol treatment. Induction of *Vdr* expression is in line with the recent observation that active Vdr signaling regulates the Vdr expression in a positive feed-back loop [37]. The increase in *Vdr* may also account for its sustained signaling activity during the medication-free period (see elevated *Cyp24a1* expression levels at 12 weeks in Figure 2(c)).

Moreover, the calcitriol treatment resulted in inhibition of Hh signaling activity as revealed by decreased *Gli1* levels (Figure 2(c)). Although calcitriol-mediated inhibition of *Gli1* expression was observed at the end of therapy, it became significant after the medication-free period (see decreased *Gli1* expression at 12 weeks in Figure 2(c)). Thus, similar to *Vdr* expression, significant downregulation of *Gli1* rather seems to be a delayed effect of calcitriol. Late effects also included the induction of the muscle differentiation markers *MyoD*, *MRF4*, and *p27* (Figure 2(d)). Although a trend in upregulation of these markers was already seen after end of therapy, it became significant after the 4-week observation period (Figure 2(d)).

Finally, calcitriol significantly inhibited the proliferative capacity of the tumors as revealed by a decrease in Ki67^+^ cells by 48% and 72% at therapy end and after the additional observation period, respectively (Figure 2(e)). An increased activity of the effector caspase 3 was not detected (data not shown).

At the first glance, the significant 48% decrease of Ki67^+^ cells directly at therapy end is in contrast with the sustained ERMS growth as measured by VCT (see “8 weeks” values in Figures 2(b) and 2(e)). However, it is well known that computer-assisted tumor size measurements have limitations. For example, this method is inaccurate in differentiating viable tumor from fibrotic or necrotic tissue. Consequently, the degree of response may be underestimated. Computer tomography is also limited in measuring responses in tumors that do not change in size during therapy [38]. This is the case in our present study. Thus, besides the significant decrease of Ki67^+^ cells, significant activation of Vdr signaling (see “8 weeks” value in Figure 2(c)) indicated a molecular effect of calcitriol, despite lack of tumor size reduction. In addition, a moderate decrease in Hh signaling activity and an increase in muscle differentiation were already detectable at therapy end.

Interestingly, decrease of Hh signaling activity and increase of muscle differentiation became significant after the medication-free observation period, when ERMS growth stagnated and when Ki67^+^ tumor cells were reduced by 72% (see “8 + 4 weeks” values in Figures 2(b) and 2(e)). This suggests that both inhibition of the Hh signaling cascade and differentiation processes are required for a measurable growth inhibition of ERMS. Activity of Hh signaling and differentiation are however unrelated to each other, because application of the specific Hh inhibitor cyclopamine does not impact ERMS differentiation (see Figure 1(d) and *Ecke *et al. [15] for *in vitro* and *in vivo* experiments, resp.).

Our data also implicate that most of the calcitriol-mediated effects (i.e., induction of differentiation, inhibition of Hh signalling, and tumor growth) are not immediate but rather late tumor responses. The reason for this phenomenon is currently unknown. One explanation could be the fact that ERMS of *Ptch* ^neo67/+^ mice are slowly growing tumors. This may render them fairly resistant to molecular changes that requir for example, cell cycle progression. Furthermore, the lack of an immediate tumor growth response suggests that calcitriol has no acute cytotoxic effects on the tumor cells. Finally, the relatively poor *in vivo* effects of calcitriol in the present study may also be due to underdosing of calcitriol. This assumption arises from our recent study using 100 ng calcitriol/kg daily for 8 weeks in the treatment of *Ptch*-associated BCC in mice [18]. As in ERMS, calcitriol led to a significant reduction of Hh signaling activity and to an induction of Vdr signaling in BCC. Calcitriol also provoked a significant decrease in Ki67^+^ cells and the expression of relevant differentiation markers. Thus, these molecular changes are general features of calcitriol and not a characteristic of the respective tumor entity. However, in contrast to the present study, calcitriol resulted in a strong and significant growth inhibition of BCC [18]. Therefore, it is certainly important to repeat the ERMS study using calcitriol at a dose of 100 ng/kg/d, which is well tolerated by mice [18]. An elongation of the treatment period (e.g., 12 or 16 weeks) may be beneficial as well, because most of the calcitriol-mediated changes seem to be late effects in ERMS.

## 4. Conclusions

Despite improvements over the last decades in the therapy of RMS the survival rate for metastatic RMS is still low. Thus new treatment options especially for patients with recurrent or metastatic RMS are needed. Our present study demonstrates that the application of the active form of vitamin D_3_, calcitriol, activates Vdr signaling and differentiation and additionally inhibits Hh signaling and proliferation of ERMS tumor cells *in vitro* and *in vivo*. Thus calcitriol could be beneficial in the treatment of these tumors. Particularly RMS showing aberrant Hh signaling activity may be responsive to calcitriol.
